# Adsorbate-induced lifting of substrate relaxation is a general mechanism governing titania surface chemistry

**DOI:** 10.1038/ncomms12888

**Published:** 2016-09-30

**Authors:** David Silber, Piotr M. Kowalski, Franziska Traeger, Maria Buchholz, Fabian Bebensee, Bernd Meyer, Christof Wöll

**Affiliations:** 1 Institute of Functional Interfaces (IFG), Karlsruhe Institute of Technology (KIT), Hermann-von-Helmholtz-Platz 1, 76344 Eggenstein-Leopoldshafen, Germany; 2 Institute of Energy and Climate Research (IEK-6), Forschungszentrum Jülich, Wilhelm-Johnen-Strasse, 52425 Jülich, Germany; 3 Westfälische Hochschule, FB 8, August-Schmidt-Ring 10, 45665 Recklinghausen, Germany; 4 Interdisciplinary Center for Molecular Materials (ICMM) and Computer-Chemistry-Center (CCC), Friedrich-Alexander-Universität Erlangen-Nürnberg, Nägelsbachstrasse 25, 91052 Erlangen, Germany

## Abstract

Under ambient conditions, almost all metals are coated by an oxide. These coatings, the result of a chemical reaction, are not passive. Many of them bind, activate and modify adsorbed molecules, processes that are exploited, for example, in heterogeneous catalysis and photochemistry. Here we report an effect of general importance that governs the bonding, structure formation and dissociation of molecules on oxidic substrates. For a specific example, methanol adsorbed on the rutile TiO_2_(110) single crystal surface, we demonstrate by using a combination of experimental and theoretical techniques that strongly bonding adsorbates can lift surface relaxations beyond their adsorption site, which leads to a significant substrate-mediated interaction between adsorbates. The result is a complex superstructure consisting of pairs of methanol molecules and unoccupied adsorption sites. Infrared spectroscopy reveals that the paired methanol molecules remain intact and do not deprotonate on the defect-free terraces of the rutile TiO_2_(110) surface.

## Abstract (web summary)

Molecules on a metal surface may be modified by the presence of oxide layers, but further mechanistic understanding is still required. Here the authors show for methanol on rutile TiO_2_(110) that strongly bonded adsorbates lift surface relaxations, leading to substrate-mediated interaction between adsorbates.

## Graphical abstract

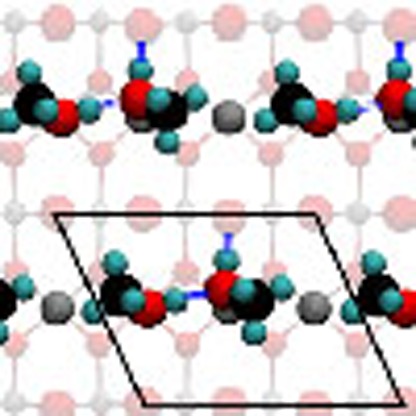

A thorough characterization of chemical processes occurring at oxide surfaces and the deduction of governing principles are presently among the most severe challenges in surface science. Gathering more detailed insights into the adsorption and subsequent reactions of molecules on oxide substrates is a matter of utmost importance, not only for heterogeneous catalysis, but also for photochemistry, photovoltaics and medicine—all metals (except gold) used in medical implants are covered by an oxide layer. Among the many different oxides of technological relevance, titania takes a special role since presently the (110) surface of rutile, the most common modification of titania, is generally considered to be the oxide substrate which is understood best, both experimentally and theoretically^1^. Despite this progress, the available experimental information on how molecules bind to this important surface is incomplete and discussed controversially, even for small adsorbates like water^2^,^3^, and simple alcohols like methanol^4^,^5^,^6^. Also on the theoretical side, the proper description of molecule/rutile interactions represents a major challenge^7^. As a result of this lack of information, the application of the so-called ‘Surface Science Approach' to unravel guiding principle in surface chemistry, which has been so successful for reactions occurring on metals^8^, has had only limited success in the case of oxides.

A particularly interesting case is methanol. Since the interaction of this smallest alcohol with TiO_2_ surfaces is important for a number of applications in heterogeneous catalysis and also in photochemistry, for example, the photoinduced generation of hydrogen by splitting of water^9^ or methanol^10^, this system has been investigated in a number of previous works. More than 15 years ago, Henderson *et al*.^11^ reported the observation of a complicated low energy electron diffraction (LEED) pattern for methanol adsorbate layers on rutile TiO_2_(110). However, until today, this LEED pattern has not been reproduced. As has been pointed out by Diebold^1^, the problem in obtaining high-quality LEED data^12^ for adsorbate layers on oxides may result from the fact that the cross-section for electron-induced decomposition and desorption is fairly large. The same problem also hampers investigations of the question whether molecules dissociate upon adsorption or stay intact. For methanol on rutile TiO_2_(110), a general consensus has not been reached yet, neither from experiment^5^,^11^,^12^,^13^,^14^,^15^ nor from theory^4^,^16^,^17^,^18^. Thus, for a reliable characterization of the structure of adsorbate layers on oxide surfaces such as rutile TiO_2_(110), which is also an active photocatalyst, methods that perturb the adsorbate layer as little as possible (that is, where electronic excitations are avoided) should be applied.

In this article we demonstrate that for methanol on TiO_2_(110) a fundamental mechanism governing the adsorption of strongly interacting molecules with oxides comes into play, which is absent for metal substrates and which has not yet been discussed on a quantitative level for ooxides, to the best of our knowledge. Using density-functional theory (DFT) calculations, we show that strongly interacting adsorbates can locally lift the relaxation present on the pristine surface beyond their immediate adsorption site. This adsorbate-induced substrate relaxation leads to a substantial reduction of binding energies for further molecules. This effective repulsion between adsorbates gives rise to a complex adsorbate structure and, importantly, prevents the dissociation of the molecules. To avoid the above-mentioned problems possibly arising from beam-damage, we use two experimental methods where such unwanted effects can be strictly excluded. The first one is the scattering of thermal-energy (less than 80 meV) He atoms. The second method is infrared (IR) reflection-absorption spectroscopy (IRRAS), which is applied to this adsorbate system for the first time, to the best of our knowledge.

## Results

### He-atom scattering

**Figure 1:**
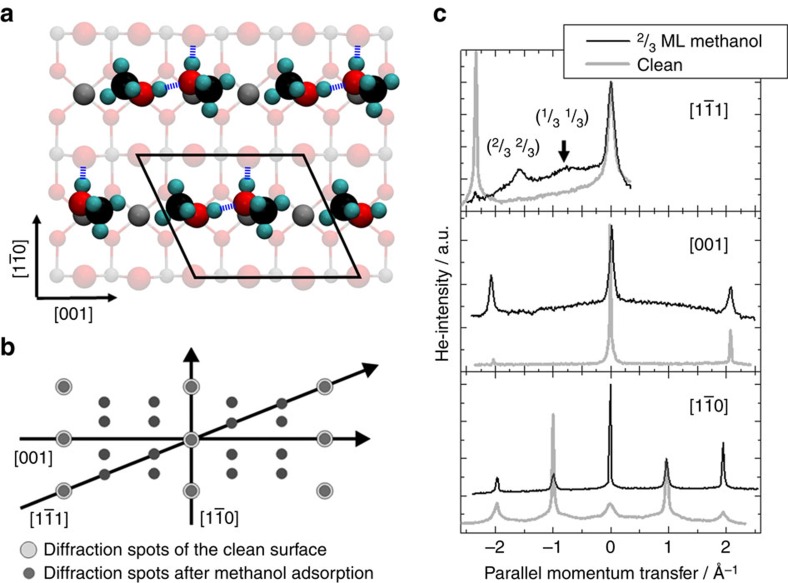
Atomic structure of the methanol layer. (**a**) Energetically most favourable layer of intact methanol molecules at ^2^/_3_ ML surface coverage as obtained from the DFT calculations. Ti, O, C and H atoms are shown in grey, red, black and cyan. The L(1 × 3) unit cell is indicated by solid lines. Consecutive [001]-rows are shifted by one lattice constant along [001] due to substrate-mediated repulsions between molecules along the 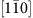-direction. (**b**) Diffraction pattern of a L(1 × 3) unit cell. (**c**) HAS angular distributions of the clean and methanol-covered surface reveal a L(1 × 3) unit cell.

After adsorption of methanol on rutile TiO_2_(110), we observe a well-defined, highly reproducible, complex diffraction pattern. In accord with previous work^11^, we propose a tilted repeat unit with size of three surface unit cells. We term this structure L(1 × 3), with ‘L' standing for ‘leaning', see Fig. 1. This finding suggests a rather complicated, unusual packing of molecules within the unit cell. In particular, the absence of any additional diffraction peak along the [001]-direction (see Fig. 1) allows to exclude the presence of a simple (1 × 3) structure.

### Density functional theory

To unravel the reason for this unexpected packing of the methanol or methoxy species on the surface, we have carried out an extensive set of DFT calculations probing more than 70 adsorbate configurations (see Supplementary Figs 1–5 and Supplementary Table 1 in the Supplementary Material). First, a single methanol molecule was adsorbed on the perfect surface using a large (2 × 4) surface unit cell. In this low coverage limit (1/8 ML), dissociation (D) is favoured: the proton from the methanol OH-group is transferred to an adjacent substrate O atom. The calculated binding energy of 0.93 eV is considerably higher than that for the molecularly intact adsorbed species (M) of 0.79 eV. In the next step, pairs of molecules, either aligned along the rows (that is, along [001]) or along the 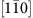-direction, were considered in the (2 × 4) unit cell. The energies of the most favourable configurations are provided in Table 1. Also in this case, dissociated species (DD) are energetically more favourable than pairs of intact molecules (MM). Within the [001]-rows, however, partially dissociated MD pairs are even more stable than the fully dissociated dimer, DD. Such MD pairs are also found to be the lowest-energy configuration of water adsorbed on ZnO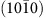 (ref. 19) although water MM dimers are only slightly higher in energy^20^.

Interestingly, when comparing the total adsorption energy of two molecules to that for a single molecule, it is observed that the gain in binding energy upon adsorbing the second molecule is substantially reduced. Further analysis showed that the reason for this unexpected finding is rather important and of general importance with regard to the interactions of adsorbate species with TiO_2_ substrates. The reduced adsorption energy does not result from a direct repulsive adsorbate–adsorbate interaction, as one might naively conclude, but has its origin in a substrate-mediated repulsion caused by an adsorption-induced re-relaxation of the titania substrate. This important effect can be understood as follows.

**Figure 2:**
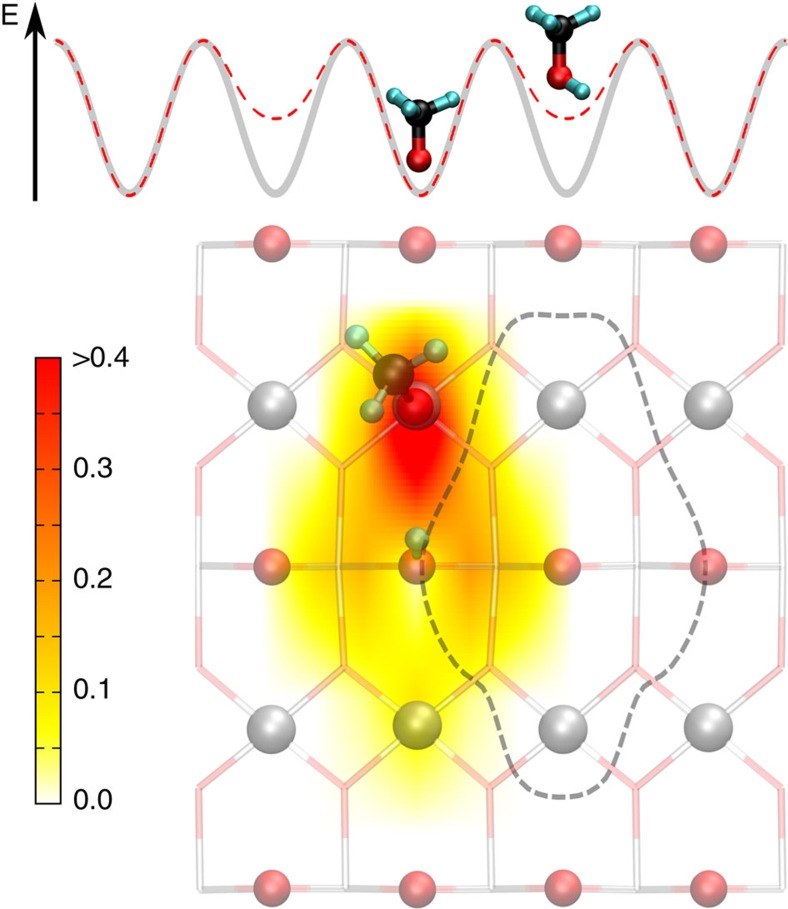
Footprint of an adsorbed methanol molecule on the rutile TiO_2_(110) surface. The potential energy diagram illustrates the effect of substrate-mediated repulsion. The energy gain for a molecule adsorbing at a clean surface (grey line) is larger than for a molecule adsorbing next to an already present adsorbate (red dashed line). The amplitude of the adsorbate-induced lifting of the titania surface relaxation, which is responsible for the effective adsorbate–adsorbate repulsion, is shown colour-coded (in Å) in the lower panel.

First, one has to take into account that the atomic positions on a clean, adsorbate-free rutile TiO_2_(110) surface are substantially different from that expected on the basis of the bulk structure. It has taken researchers a severe effort to correctly reproduce experimental X-ray diffraction data^21^ for the rutile TiO_2_(110) surface with DFT calculations^22^. Briefly, the surface atoms strongly relax inward, leading to an energy gain of about 1.04 eV per surface unit cell^7^. This substantial surface relaxation is to some extent lifted upon forming bonds to the adsorbed methanol. It is instructive to decompose this re-relaxation into two steps. First, we consider the rather substantial rearrangement of the substrate atoms, which requires the energy of 2.66 eV. This energetically very costly ‘bond preparation' of the titania substrate is, however, compensated by the large gain in binding energy of the methoxy species, which leads to an overall stabilization of 0.93 eV. In fact, if the substrate atoms are kept frozen in the geometry optimization, single dissociated methanol molecules are unstable and recombine spontaneously. Since the adsorption-induced re-relaxation extends well beyond the unit cell containing the adsorbed molecule (see Fig. 2), the full gain in adsorption energy is not available to neighbouring adsorbates, because at adjacent sites the substrate underneath is already partially re-relaxed. The re-relaxation thus leads to an effective adsorbate–adsorbate repulsion.

The substrate-mediated repulsion is most pronounced for molecules aligned along the [001]-direction and strongest for pairs of methoxy species (DD), see Table 1. In contrast, MD and MM pairs gain additional binding energy by forming H-bonds between the neighbouring molecules (see Fig. 1). These intermolecular H-bonds are stronger than those to the oxygen anions of the surface. This direct attractive adsorbate–adsorbate interaction contributes to the stabilization of MD and MM compared with DD pairs. This insight, gained from a detailed investigation of binding energies of isolated pairs, is important, since it allows to predict that a turnover from fully dissociative to partially dissociative or even molecular intact adsorption will occur for higher coverage, when the larger substrate-mediated effective adsorbate–adsorbate repulsion between dissociated molecules makes dissociation increasingly unfavourable.

Using the information gained for the low coverage limit, we will now address the structure of a full (saturated) monolayer. The different pairs of [001]-aligned methanol molecules (discussed above in the low-coverage limit) were placed into a (1 × 2) surface unit cell and a full structural optimization was performed. The calculated binding energies in Table 1 show that at the 1 ML coverage strong steric repulsions between the methanol molecules are present, since the nearest-neighbour distance between the Ti_5c_ adsorption sites is too short for the steric demand of the methyl groups (see Fig. 1). To avoid this steric repulsion, one lattice site between the methanol pairs needs to remain empty. Indeed, for pairs of methanol molecules in a (1 × 3) unit cell. much larger binding energies per molecule are obtained (see Table 1). Inspection of Table 1 also reveals that, as expected, the larger substrate-mediated repulsion for dissociated molecules makes full dissociation the least favourable adsorption mode.

The substrate-mediated adsorbate repulsion is also responsible for the experimentally observed tilt of the (1 × 3) unit cell. The calculations for isolated methanol pairs in the (2 × 4) unit cell show that the repulsion between molecules also extends along the 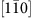-direction (see Table 1). A shift of consecutive [001]-rows consisting of methanol pairs and empty adsorption sites by one lattice constant along the [001]-direction, leading to a L(1 × 3) periodicity (see Fig. 1), should therefore result in a further stabilization of the adsorbate layer. Indeed, DFT calculations show that the structure with a tilted unit cell is more stable than the (1 × 3) layer by 0.02 eV.

### Phase diagram

**Figure 3:**
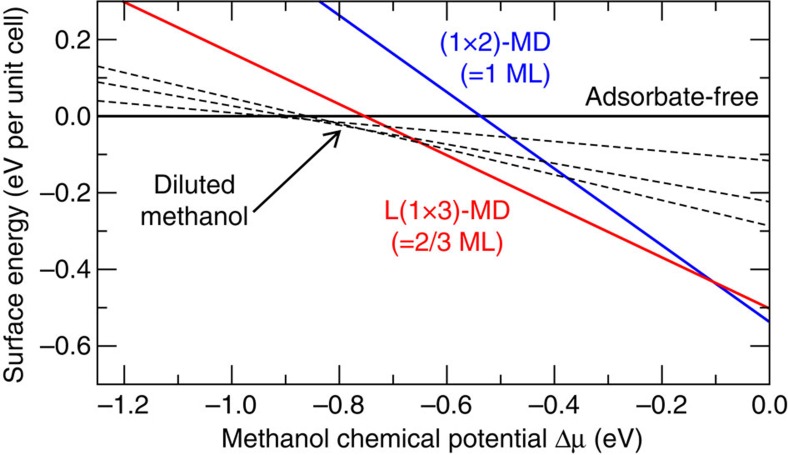
Surface phase diagram. Thermodynamic stability of the adsorbate-free and various methanol-covered rutile TiO_2_(110) surfaces as function of the methanol chemical potential of a surrounding gas phase.

To confirm that indeed ^2^/_3_ ML is the preferred methanol coverage on rutile TiO_2_(110), we analysed the thermodynamic stability of the methanol layers by converting the calculated binding energies per molecule into changes of the surface energy per surface area^23^. The experimental temperature and methanol partial-pressure conditions are represented by a chemical potential. The surface phase diagram in Fig. 3 shows that the L(1 × 3) structure with ^2^/_3_ ML coverage is the thermodynamically most stable configuration over a wide range of temperature and pressure conditions. Full monolayer coverage will only form at rather high chemical potential (low temperature, high pressure). With increasing temperature, methanol starts to desorb and for an intermediate temperature range some diluted methanol molecules may be found on the surface before the surface becomes adsorbate-free.

Unfortunately, it is difficult to answer the question on whether methanol stays intact or dissociates (deprotonates) upon adsorption solely on the basis of the DFT calculations, since the MM and MD structures are very close in energy. In particular, careful convergence tests (see Supplementary Figs 1–3) show that molecular adsorption (MM) and partial dissociation (MD) become energetically equivalent with increasing thickness of the rutile TiO_2_(110) slab. As extrapolated binding energy of methanol in the L(1 × 3) structure for infinite slab thickness we obtain 0.72 eV.

### IR spectroscopy

**Figure 4:**
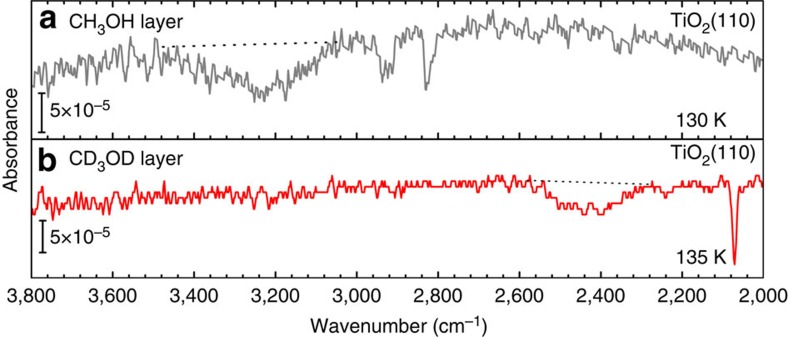
IR reflectance-adsorption spectra. (**a**) Methanol (CH_3_OH) and (**b**) deuterated methanol (CD_3_OD) adsorbate layer on the rutile TiO_2_(110) surface.

To decide unequivocally whether methanol dissociates on rutile TiO_2_(110) or not, we have performed measurements using IRRAS. The data shown in Fig. 4 for a methanol-saturated TiO_2_(110) surface reveal the presence of a broad OH-stretch band with full width at half maximum (FWHM) of about 200 cm^−1^ centred at 3,300 cm^−1^, which is shifted to 2,500 cm^−1^ (with a FWHM∼150 cm^−1^) when using deuterated CD_3_OD methanol. From the absence of a band at 3,710 cm^−1^, which would be characteristic for vibrations of OH species resulting from a transfer of protons to substrate O atoms^24^, we conclude that all methanol species within the L(1 × 3) structure must be intact (comparison with this previous work^24^ yields an upper bound for the amount of dissociated molecules of 0.01 ML, see Supplementary Note 1). This conclusion is nicely corroborated by the calculated vibrational frequencies in the energetically most favourable structure for non-dissociated methanol. For the MM species in this structure we yield an OH-stretch vibration of 3,261 cm^−1^ (see Supplementary Table 3). The pronounced broadening present in the experimental data is typical for OH-species which are H-bonded to neighbouring O atoms^25^. For a full assignment of the other bands occurring in the IR spectra, see the Supplementary Fig. 7.

### Other adsorbates on rutile TiO_2_(110)

Although we expect that the effect described here, adsorbate-induced lifting of substrate relaxation leading to an effective adsorbate-adsorbate repulsion, is of general importance for oxides, systematic investigations of other, larger molecular adsorbates on rutile TiO_2_(110) and other substrates are beyond the scope of the present work. It is instructive, however, to compare with the case of water adsorbed on this surface. In our previous DFT calculations^7^, we found that for water the surface re-relaxations are of the same magnitude as for methanol and the induced repulsion between neighbouring water molecules is of similar strength. Thus, the same transition from a preferred dissociation of single molecules to a molecular adsorption at increasing coverage is observed. However, in contrast to methanol, the smaller water molecule can form a second H-bond to its neighbours. While the steric hindrance between the methyl groups limits the methanol coverage to ^2^/_3_ ML, water can form a full monolayer of intact molecules with a simple (1 × 1) periodicity^7^. The stabilization by the additional H-bonds leads to a slightly higher binding energy of 0.82 eV (ref. 7). Both, the presence of a (1 × 1) structure and a binding energy of 0.82 eV for water on rutile TiO_2_(110) have been confirmed by recent experiments^26^.

## Discussion

Our detailed experimental and theoretical investigation on saturated, highly ordered layer of methanol on rutile TiO_2_(110) reveals that a structure with a tilted L(1 × 3) unit cell containing two intact methanol molecules is in good agreement with all available experimental findings. This structure corresponds to a coverage of ^2^/_3_ ML and a binding energy of 0.72 eV per molecule, in excellent agreement with the results of the careful TDS study carried out by Li *et al*.^27^ and is also consistent with the LEED pattern observed by Henderson *et al*.^11^ as well as the He-atom scattering (HAS) diffraction data reported here. Note that the structure shown in Fig. 1 is energetically almost degenerate with three other configurations, which differ only by the orientation of the methyl group of the methanol molecule (up or down) or the tilt of the unit cell (left or right). This success reveals that even complicated superstructures formed on rutile substrates may be unravelled by combining high-level DFT calculations with appropriate experimental techniques. The analysis of the calculations allows identifying two important mechanisms governing adsorption on TiO_2_ substrates. While interaction of a single molecule with the substrate favours dissociation, substrate-mediated repulsion by lifting of surface relaxations together with the formation of intermolecular H-bonds leads to the stabilization of pairs of intact molecules, yielding a rather complicated L(1 × 3) unit cell. We believe that this balance between strong interaction with the oxide substrate and the corresponding lifting of surface relaxations giving rise to a substrate-mediated repulsion is a rather general phenomenon, which has to be generally considered for the interaction of more reactive molecules with oxide surfaces and for catalytic processes on such substrates.

## Methods

### HAS experiments

The He-atom scattering set-up used in these experiments as well as the HAS technique have been described in detail previously^28^. The present set-up was recently used to determine a (1 × 1) adsorption geometry for water adsorbed on rutile TiO_2_(110) (ref. 26). The probing beam has kinetic energies between *E*_i_=10 and 80 meV and consists of neutral He atoms to avoid beam damage. The FWHM of the beam energy amounts to about 2%. The scattering conditions and symmetry directions (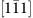, [001] and 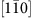) were varied by rotating the single crystal around an axis normal to the scattering plane. The resolution of the angular distributions in this investigation amounted to 0.1°. The angular distributions are presented here as a function of parallel momentum transfer.

### IR spectroscopy

An advanced UHV system (Prevac, Poland) equipped with an IR spectrometer (Bruker Vertex 80v, Germany) and several other surface-sensitive techniques (XPS and LEED) has been used for the IR experiments. A four-axis manipulator allowed to record polarization-dependent IR spectra in reflection for different azimuths of the substrate (for details, see ref. 29).

The rutile TiO_2_(110) single crystals (CrysTec, Germany) used in these experiments were cleaned by Ar^+^-sputtering (1 × 10^−6^ mbar, 10 min) and annealing at 800 K for 10 min. The cleanliness and oxidation states of the sample were monitored by X-ray photoelectron spectroscopy and the cleaning procedure was repeated several times till no carbon contamination was observed. In addition, the structural quality was checked by HAS. IRRAS measurements were only conducted after a well-defined (1 × 1) LEED pattern has been observed.

Methanol (purity 99.97 vol%, Linde, Germany) was dosed via backfilling the UHV-IR-chamber up to 10^−8^ mbar at sample temperatures typically below RT. Typically, for one spectrum, 1,024 scans were accumulated at the corresponding temperature (in the range 60 K–130 K), with the resolution set to 4 cm^−1^. All spectra shown are difference spectra obtained by subtracting a spectrum recorded for the clean TiO_2_ substrate immediately before the dosing procedure.

### DFT calculations

The periodic plane wave DFT calculations were performed using the PWscf code of the Quantum Espresso program package^30^. The Perdew–Burke–Ernzerhof PBE exchange-correlation functional was applied together with Vanderbilt ultrasoft pseudopotentials and a plane-wave cutoff energy of 30 Ry. Previous studies^7^,^19^ have shown that this setup gives reliable and accurate results for the strength of hydrogen bonds and the interaction of water with metal oxide surfaces. Surface structures were represented by periodic slabs with a thickness of five triple layers. The bottom of the slabs was passivated with pseudo-hydrogen atoms as described in ref. 7. Each configuration was relaxed by minimizing the atomic forces below a threshold of 5 meV Å^−1^. The atoms in the bottom two triple layers were constrained to their equilibrium bulk positions. The k-point density for Brillouin-zone integrations was (4,8,1) per primitive surface unit cell or higher. The analysis of the thermodynamic stability of the different methanol coverage was done as described in ref. 23.

### Data availability

The data that support the findings of this study are available from the corresponding author upon request.

## Additional information

**How to cite this article:** Silber, D. *et al*. Adsorbate-induced lifting of substrate relaxation is a general mechanism governing titania surface chemistry. *Nat. Commun.* 7:12888 doi: 10.1038/ncomms12888 (2016).

## Supplementary Material

Supplementary Information Supplementary Figures 1-7, Supplementary Tables 1-3, Supplementary Note 1 and Supplementary References.

## Figures and Tables

**Table 1:** Binding energies (in eV per molecule) for pairs of methanol molecules in different arrangements on the rutile TiO_2_(110) surface.

	**Pairs along [001]**	**Pairs along** 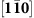	**2/3 ML (1 × 3)**	**1 ML (1 × 2)**
(DD)	0.797 (0.130)	0.892 (0.035)	0.677 (0.250)	0.440 (0.486)
(MD)	0.812 (0.048)	0.832 (0.027)	0.753 (0.106)	0.537 (0.322)
(MM)	0.773 (0.019)	0.765 (0.027)	0.719 (0.072)	0.476 (0.316)

The repulsion energy between the methanol molecules (in eV) is given in brackets. The binding energy for single molecules is 0.927 eV (D) and 0.792 eV (M). (D, dissociated; M, molecular).
