# Supplementary material for: Adsorbate-induced lifting of substrate relaxation is a general mechanism governing titania surface chemistry
Source: Nat Commun. 2016 Sep 30;7:12888. doi: 10.1038/ncomms12888 (PMC5056433; doi:10.1038/ncomms12888)
Supplement: Supplementary Information — Supplementary Figures 1-7, Supplementary Tables 1-3, Supplementary Note 1 and Supplementary References. [file ncomms12888-s1.pdf]

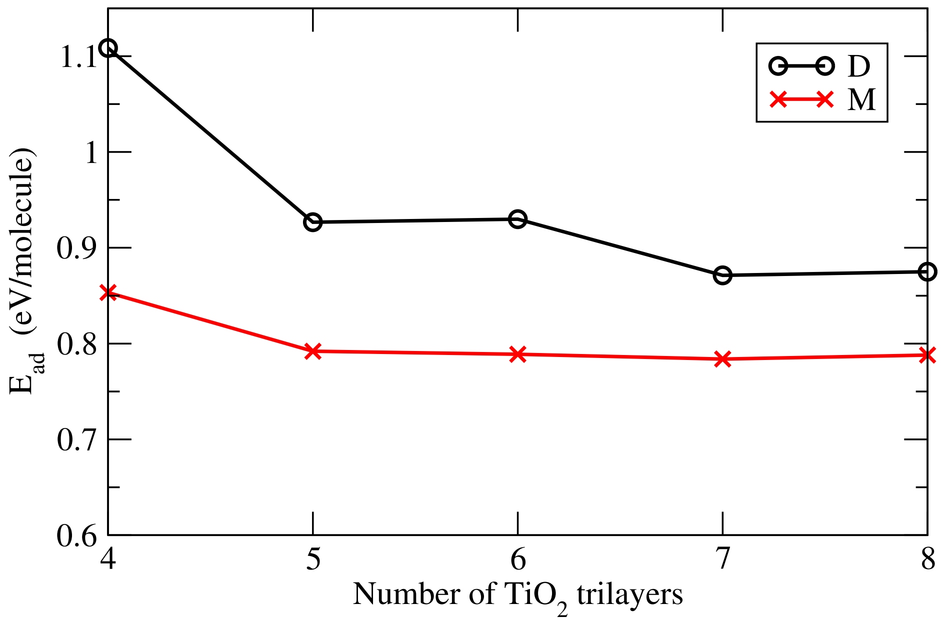


**Supplementary Figure 1 | Convergence test for adsorption of single methanol molecules.** Adsorption energy $E_{\mathrm{ad}}$ for dissociative (D) and molecular (M) adsorption of single methanol molecules in a (2×4) surface unit cell with increasing slab thickness. The atoms in the lower half of the slab were kept fixed at their bulk positions. The bottom of the slabs was passivated with pseudo-hydrogen atoms. This reduces significantly the oscillations of $E_{\mathrm{ad}}$ with slab thickness. The 5-trilayer calculations deviate by less than 0.04 eV (D) and 0.01 eV (M) from the extrapolated results for infinite slab thickness.

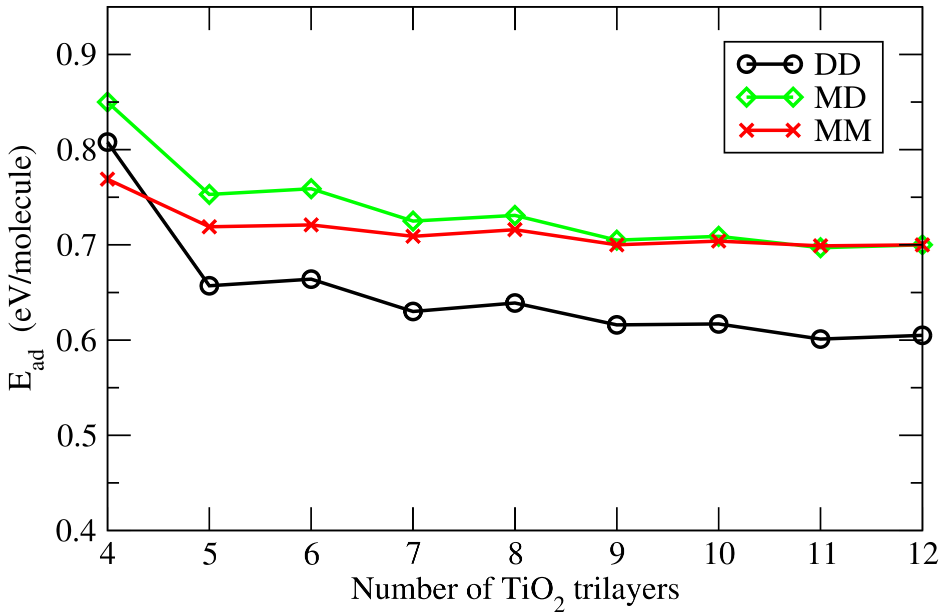


**Supplementary Figure 2 | Convergence test at 2/3 monolayer coverage.** Adsorption energy $E_{\text{ad}}$ per molecule for dissociative (DD), mixed (MD) and molecular (MM) adsorption of pairs of methanol molecules in a (1×3) surface unit cell with increasing slab thickness. The same setup as in the calculations for Supplementary Figure 1 was used. The 5-trilayer calculations deviate by less than 0.05 eV (DD), 0.04 eV (MD) and 0.02 eV (MM) from the extrapolated results for infinite slab thickness.

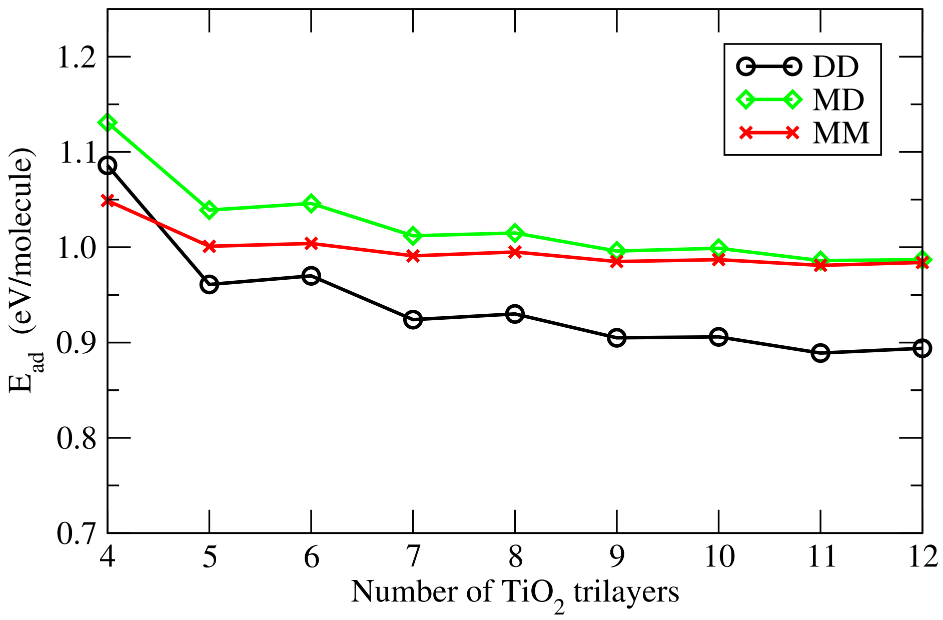


**Supplementary Figure 3 | Influence of van der Waals corrections.** Same as Supplementary Figure 2, but the calculations were performed including the Grimme D3 dispersion correction scheme[1] with the Becke-Johnson damping function[2] and dispersion coefficients specifically derived for $TiO_2$ (see Ref.[3]). The energy differences between DD, MD and MM adsorption are not significantly modified.

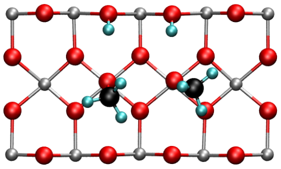
(a) DD: $E_{ad}$=0.775 eV

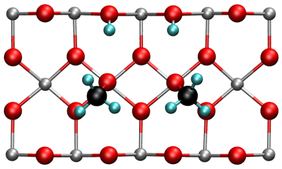
(b) DD: $E_{ad}$=0.770 eV

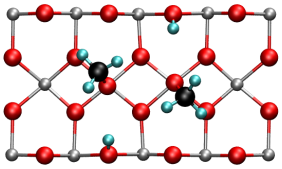
(c) DD: $E_{ad}$=0.797 eV

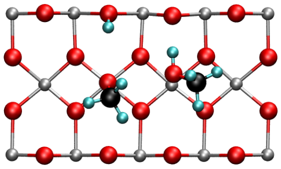
(d) DM: $E_{ad}$=0.789 eV

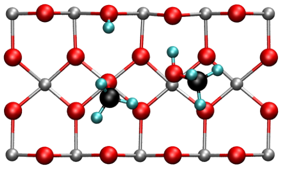
(e) DM: $E_{ad}$=0.791 eV

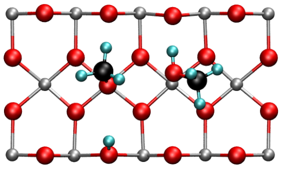
(f) DM: $E_{ad}$=0.795 eV

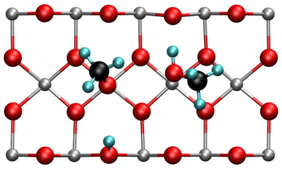
(g) DM: $E_{ad}$=0.795 eV

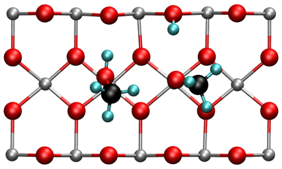
(h) MD: $E_{ad}$=0.769 eV

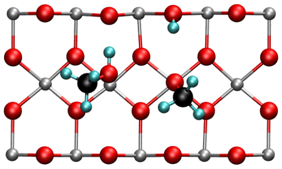
(i) MD: $E_{ad}$=0.786 eV

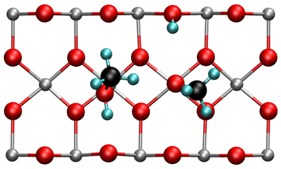
(j) MD: $E_{ad}$=0.768 eV

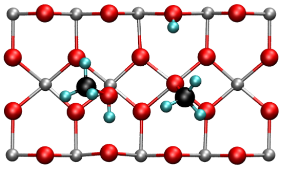
(k) MD: $E_{ad}$=0.793 eV

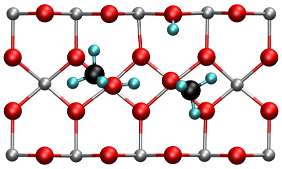
(l) MD: $E_{ad}$=0.810 eV

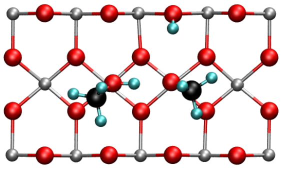
(m) MD: $E_{ad}$=0.812 eV

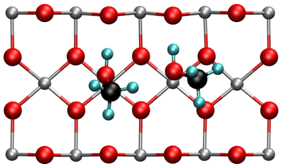
(n) MM: $E_{ad}$=0.731 eV

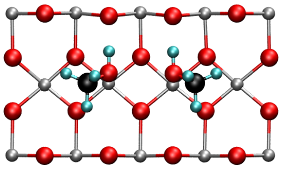
(o) MM: $E_{ad}$=0.740 eV

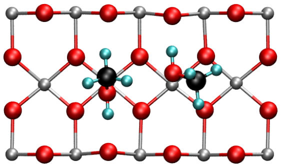
(p) MM: $E_{ad}$=0.730 eV

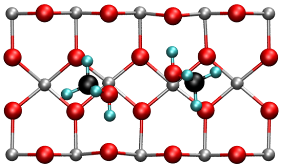
(q) MM: $E_{ad}$=0.754 eV

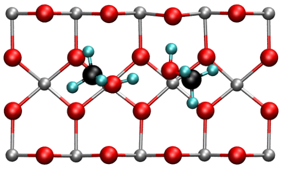
(r) MM: $E_{ad}$=0.773 eV

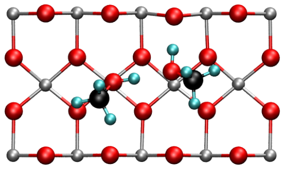
(s) MM: $E_{ad}$=0.766 eV

**Supplementary Figure 4 | Methanol pairs at 1/4 monolayer coverage.** Top view of the relaxed atomic structure of adsorbed pairs of methanol molecules on rutile $TiO_2$(110) using a (2×4) surface unit cell. This corresponds to a methanol surface coverage of 1/4 monolayer. Ti, O, C and H atoms are shown in gray, red, black and cyan, respectively.

**Supplementary Table 1 | Methanol pairs at 2/3 monolayer coverage.** Adsorption energy $E_{\mathrm{ad}}$ (in eV) per molecule for pairs of methanol molecules on rutile $TiO_2(110)$. Selected pairs from the previous calculations using a (2×4) surface unit cell (see Supplementary Figure S4) were placed in a smaller (1×3) cell. Only small structural changes were observed in the subsequent geometry optimization. The binding energy, however, decreases due to the substrate adsorption footprint of the molecules (substrate-mediated molecule–molecule repulsion by lifting of surface relaxations). The methanol binding energy increases on average by about 0.02 eV, if the (1×3) unit cell is tilted either to the left or to the right.

| Configuration | (2×4) – 1/4 ML | (1×3) – 2/3 ML |
|---|---|---|
| DD (S-Fig. 4a) | 0.775 | 0.677 |
| DD (S-Fig. 4c) | 0.797 | 0.677 |
| DM (S-Fig. 4e) | 0.791 | 0.718 |
| DM (S-Fig. 4g) | 0.795 | 0.716 |
| MD (S-Fig. 4i) | 0.786 | 0.718 |
| MD (S-Fig. 4k) | 0.793 | 0.714 |
| MD (S-Fig. 4l) | 0.810 | 0.752 |
| MD (S-Fig. 4m) | 0.812 | 0.753 |
| MM (S-Fig. 4o) | 0.740 | 0.671 |
| MM (S-Fig. 4q) | 0.754 | 0.678 |
| MM (S-Fig. 4r) | 0.773 | 0.719 |
| MM (S-Fig. 4s) | 0.766 | 0.715 |

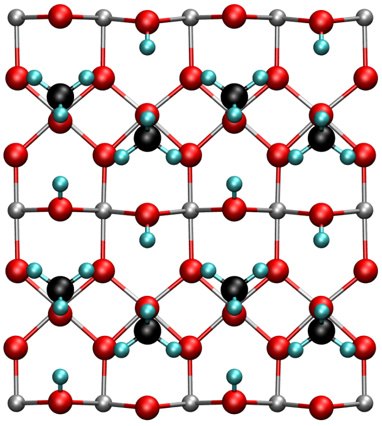

(a) DD: $E_{\mathrm{ad}}$=0.440 eV

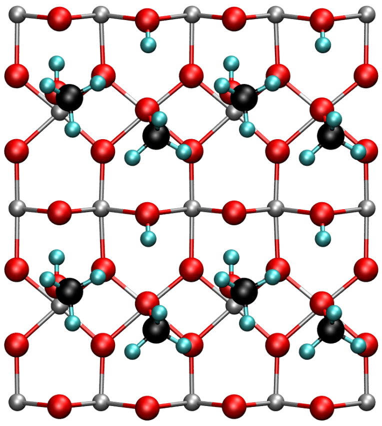

(b) MD: $E_{\mathrm{ad}}$=0.456 eV

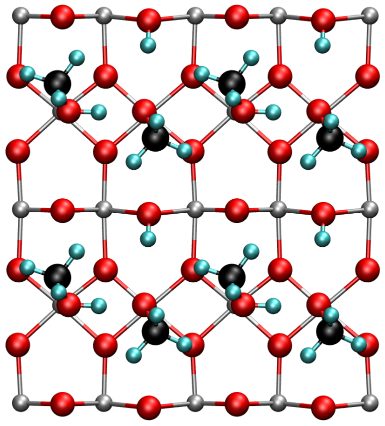

(c) MD: $E_{\mathrm{ad}}$=0.537 eV

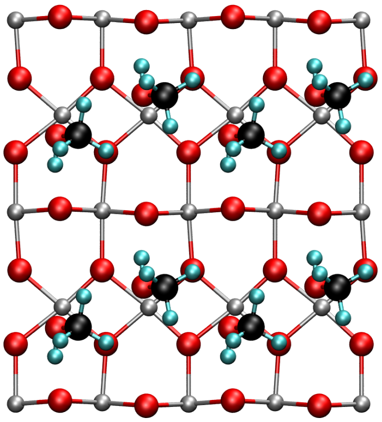

(d) MM: $E_{\mathrm{ad}}$=0.424 eV

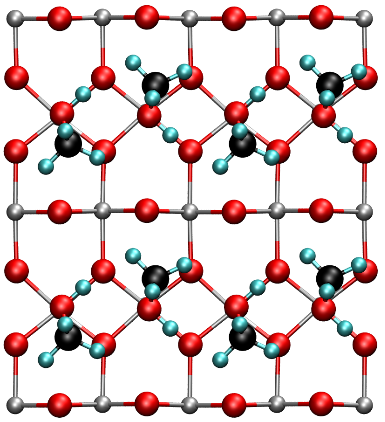

(e) MM: $E_{\mathrm{ad}}$=0.434 eV

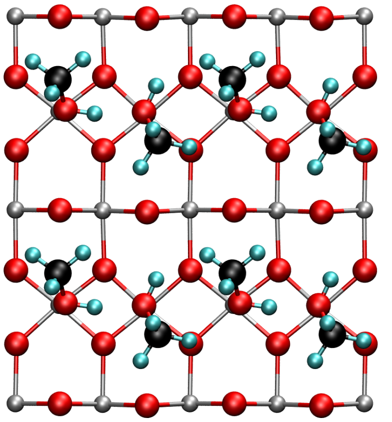

(f) MM: $E_{\mathrm{ad}}$=0.476 eV

**Supplementary Figure 5 | Methanol pairs at full monolayer coverage.** Top view of the relaxed atomic structure of adsorbed pairs of methanol molecules on rutile $TiO_2$(110) using a (1×2) surface unit cell. Steric repulsion between the molecules leads to low adsorption energies. Ti, O, C and H atoms are shown in gray, red, black and cyan, respectively.

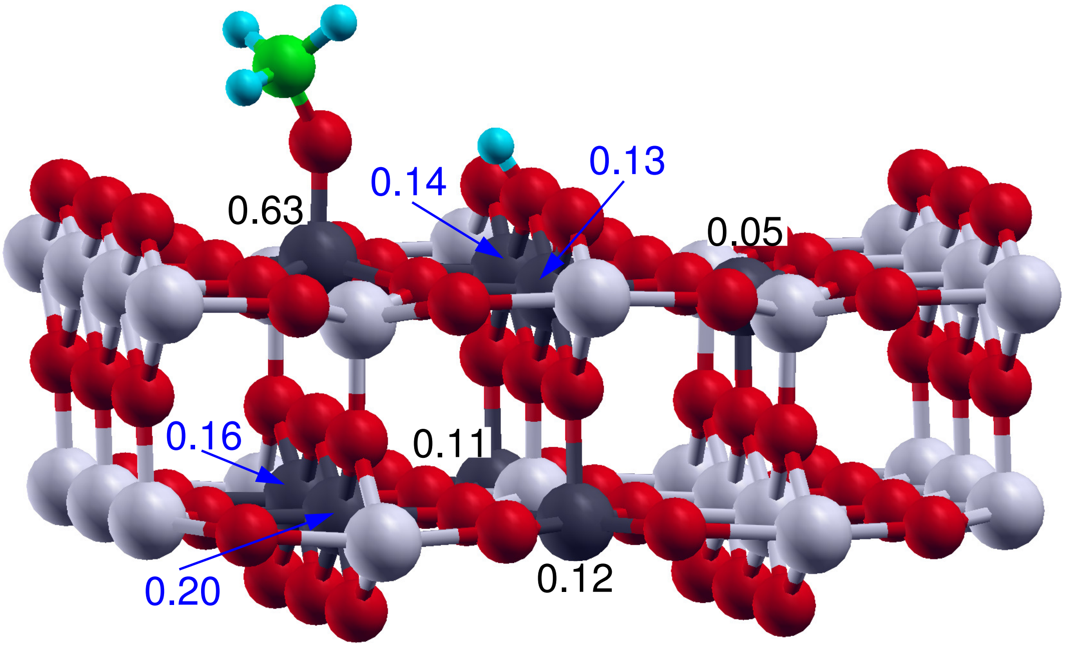


**Supplementary Figure 6 | Adsorption footprint.** Adsorbate-induced atomic relaxations of the substrate. Ti, O, C and H atoms are shown in gray, red, green and cyan, respectively. The Ti atoms with the largest displacements are indicated in black. The atomic displacements are given in Å.

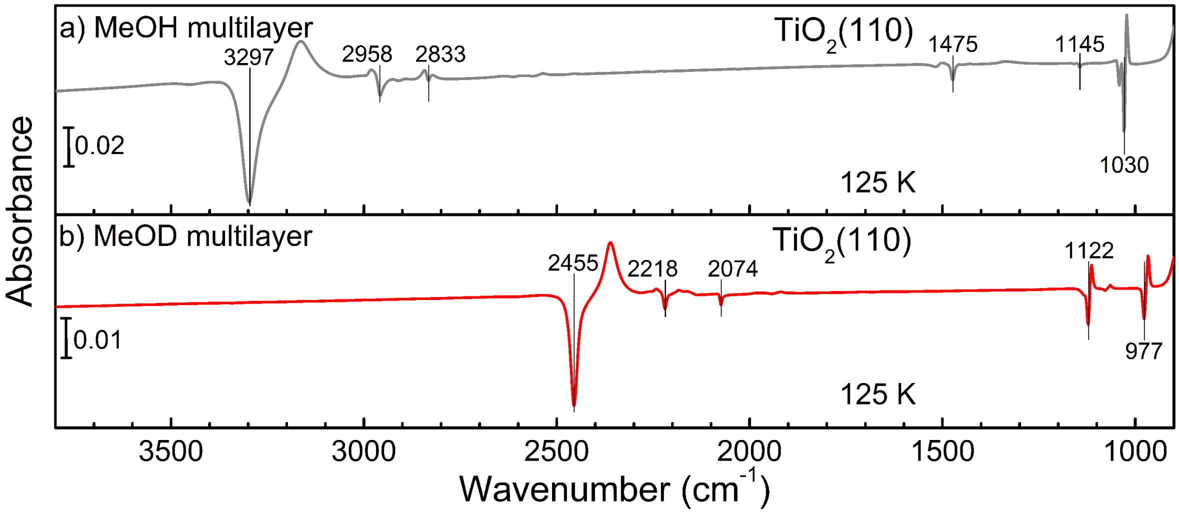


**Supplementary Figure 7 | Additional IR Measurements.** IR reflection-adsorption spectra of (a) methanol ($CH_3OH$) and (b) deuterated methanol ($CD_3OD$) multilayers on the rutile $TiO_2(110)$ surface.

**Supplementary Table 2 | Assignment of vibrational modes.** Vibrational mode assignments of methanol ($CH_3OH$) and deuterated methanol ($CD_3OD$) multilayers on the rutile $TiO_2(110)$ surface.

| $CH_3OH$ | | $CD_3OD$ | |
|---|---|---|---|
| $\nu$ (cm$^{-1}$) | Mode | $\nu$ (cm$^{-1}$) | Mode |
| 3297 | $\nu_{as}(OH)$ | 2455 | $\nu_{as}(OD)$ |
| 2958 | $\nu_{as}(CH_3)$ | 2218 | $\nu_{as}(CD_3)$ |
| 2833 | $\nu_s(CH_3)$ | 2047 | $\nu_s(CD_3)$ |
| 1475 | $\delta_s(CH_3)$ | 1122 | $\delta_s(CD_3)$ |
| 1145 | $\rho(CH_3)$ | | |
| 1030 | $\nu(CO)$ | 977 | $\nu(CO)$ |

**Supplementary Table 3 | Calculated vibrational frequencies and IR intensities.** Vibrational frequencies $\nu$ (in $cm^{-1}$) and IR intensities (in km/mol) calculated for a pair of dissociated (DD, S-Fig. 4c), mixed (MD, S-Fig. 4m) and molecular (MM, S-Fig. 4r) adsorbed methanol molecules in a L(1×3) surface unit cell.

| L(1×3)–DD | | L(1×3)–MD | | L(1×3)–MM | |
|---|---|---|---|---|---|
| $\nu$ ($cm^{-1}$) | Intensity | $\nu$ ($cm^{-1}$) | Intensity | $\nu$ ($cm^{-1}$) | Intensity |
| 760.8 | 0.94 | 832.4 | 0.86 | 999.6 | 8.50 |
| 806.9 | 0.19 | 1013.6 | 6.29 | 1024.7 | 39.25 |
| 1061.7 | 18.34 | 1041.4 | 64.64 | 1109.5 | 3.67 |
| 1104.0 | 77.53 | 1116.7 | 1.01 | 1118.0 | 4.41 |
| 1114.6 | 1.97 | 1123.2 | 0.00 | 1133.6 | 0.00 |
| 1115.3 | 1.40 | 1132.4 | 0.15 | 1135.1 | 0.18 |
| 1126.5 | 0.69 | 1134.4 | 8.15 | 1388.6 | 18.35 |
| 1129.6 | 46.64 | 1404.3 | 0.13 | 1406.3 | 5.28 |
| 1405.0 | 0.08 | 1413.5 | 1.54 | 1411.2 | 0.00 |
| 1406.1 | 0.65 | 1426.4 | 1.58 | 1419.4 | 0.47 |
| 1421.6 | 0.98 | 1432.7 | 0.01 | 1436.2 | 0.01 |
| 1429.5 | 0.27 | 1436.7 | 0.92 | 1438.5 | 0.97 |
| 1434.4 | 0.20 | 1439.5 | 0.00 | 1452.1 | 2.92 |
| 1434.6 | 1.51 | 1464.2 | 13.16 | 1459.4 | 0.18 |
| 2888.3 | 12.54 | 2899.6 | 14.00 | 2878.4[b] | 109.07 |
| 2912.7 | 32.31 | 2942.7 | 24.69 | 2942.4 | 11.51 |
| 2958.4 | 12.51 | 2970.6 | 11.36 | 2945.2 | 9.66 |
| 2984.1 | 0.10 | 2999.1 | 2.24 | 3014.1 | 7.63 |
| 2985.7 | 3.04 | 3022.0 | 2.14 | 3022.1 | 5.08 |
| 2996.7 | 1.74 | 3053.0 | 0.00 | 3045.0 | 1.43 |
| 3556.4[a] | 10.22 | 3081.8[b] | 7.77 | 3061.8 | 0.16 |
| 3629.7[a] | 11.74 | 3560.3[a] | 13.44 | 3262.1[b] | 4.19 |

[a]$HO_{surf}$ vibration [b]$HO_{meth}$ vibration

# Supplementary Note

An upper limit for the amount of dissociated methanol species (yielding an OH group by protonation of a substrate O atom) can be estimated as follows: As the basis we use the known frequency of the OH-stretch vibration on this surface as determined by IRRAS, $3710\,cm^{-1}$ (see Ref. [4]). In these previous experiments, the number of OH groups was estimated to be 0.1 ML (0.05 ML O vacancies, 2 OH groups per vacancy). Assuming the same intensity of the OH band (absorbance of $2\times10^{-4}$) as seen in the previous IR experiments and considering an upper limit of $2\times10^{-5}$ in the present experiments (see Figure 4a in the manuscript), we yield an upper limit of 0.01 ML of OH groups in our experiments. This upper limit is in line with previous estimates on the number of defects on the oxidized titania surface.

# Supplementary References


[1] S. Grimme, J. Antony, S. Ehrlich, H. Krieg, J. Chem. Phys. **132**, 154104 (2010).

[2] S. Grimme, S. Ehrlich, L. Goerigk, J. Comput. Chem. **32**, 1456 (2011).

[3] J. Moellmann, S. Ehrlich, R. Tonner, S. Grimme, J. Phys.: Condens. Matter **24**, 424206 (2012).

[4] N.G. Petrik, G.A. Kimmel, J. Phys. Chem. C **119**, 23059 (2015).
